# Postfilter for Dual Channel Speech Enhancement Using Coherence and Statistical Model-Based Noise Estimation

**DOI:** 10.3390/s24123979

**Published:** 2024-06-19

**Authors:** Sein Cheong, Minseung Kim, Jong Won Shin

**Affiliations:** School of Electrical Engineering and Computer Science, Gwangju Institute of Science and Technology, Gwangju 61005, Republic of Korea; seiinjung@gm.gist.ac.kr (S.C.); kms0603@gm.gist.ac.kr (M.K.)

**Keywords:** noise PSD estimation, coherence, dual channel speech enhancement, postfilter, speech presence probability estimation

## Abstract

A multichannel speech enhancement system usually consists of spatial filters such as adaptive beamformers followed by postfilters, which suppress remaining noise. Accurate estimation of the power spectral density (PSD) of the residual noise is crucial for successful noise reduction in the postfilters. In this paper, we propose a postfilter utilizing proposed *a posteriori* speech presence probability (SPP) and noise PSD estimators, which are based on both the coherence and the statistical models. We model the coherence-based *a posteriori* SPP as a simple function of the magnitude of coherence between two microphone signals and combine it with a single-channel SPP based on statistical models. The coherence-based estimator for the PSD of the noise remaining in the beamformer output in the presence of speech is derived using the pseudo-coherence considering the effect of the beamformers, which is used to construct the coherence-based noise PSD estimator. Then, the final noise PSD estimator is obtained by combining the coherence-based and statistical model-based noise PSD estimators with the proposed SPP. The spectral gain function is also modified, incorporating the proposed SPP. Experimental results demonstrate that the proposed method led to more accurate noise PSD estimation and perceptual evaluation of speech quality scores in various diffuse noise environments, and did not degrade the speech quality under the presence of directional interference, although the proposed method utilizes the coherence information.

## 1. Introduction

Over the past decades, there has been a growing demand for speech enhancement using microphone arrays in speech processing applications such as automatic speech recognition, mobile communications, and hearing aids [1,2,3,4]. Multichannel speech enhancement aims to reduce the additive noise and improve the quality of the speech signals obtained by multiple microphones placed in a variety of acoustic environments [5,6,7,8,9,10,11,12,13,14,15,16,17,18,19,20,21,22,23,24,25,26,27,28,29,30,31,32]. In many multichannel speech enhancement systems, beamforming algorithms, such as the minimum-variance distortionless-response (MVDR) beamformer [11] and the general transfer function generalized sidelobe canceler (TF-GSC) [12,13], have been employed to extract a desired signal, exploiting spatial information on the location of the sound sources. Although these beamformers successfully reduce the interfering noise without creating too much speech distortion, the amount of noise suppression is not very high in general, and nonstationary interferences and diffuse noises may disrupt some of the beamformers. Therefore, an additional postfilter is usually used to further enhance the output of the beamformer [14,15,16,17,18,19,20,21,22,23,24,25,26,27,28]. It has been shown that the multichannel Wiener filter (MWF) can be factorized into the MVDR beamformer and a single-channel Wiener postfilter [14,15].

The postfilters used in the literature include the Wiener filter [16,17,18,19], short-time spectral amplitude (STSA) estimator [20], and optimally modified log-spectral amplitude (OM-LSA) [21] estimator, for all of which the accurate estimation of the noise power spectral density (PSD) in the beamformer output is crucial. The noise PSD estimation approaches for the postfilter can be classified into two categories. The methods falling into the first category are essentially single-channel approaches which estimate the noise PSD from the output of the beamformer [22,23] using single-channel noise estimation approaches [33,34,35,36,37]. The advantage of these approaches is that the performance of the postfilter is not severely affected by the steering error of the beamformer. However, the single-channel noise PSD estimation approaches cannot rapidly track the changes in the noise statistics and thus, the noise PSD is underestimated for nonstationary noises, resulting in insufficient noise suppression. The methods in the second category are multichannel approaches which utilize spatial information from the microphone signals or beamformers. In [18], the noise PSD is estimated by a recursive averaging of the power spectrum of the null-beamformed signal. In [21], the noise PSD estimate is obtained by a recursive averaging of the periodogram of the beamformer output, with a smoothing factor dependent on the speech presence probability (SPP). The SPP in [21] is affected by transient beam-to-reference ratio (TBRR), which is the ratio of the transient powers in the beamformer output and the noise reference signals of the TF-GSC obtained by applying the minima controlled recursive averaging (MCRA) to those signals. These methods utilizing the beamformer output signals can effectively deal with moderately nonstationary noises, but the performance deteriorates when the steering error occurs in the beamformer or the noise is highly nonstationary. The leakage of the speech signal into the noise reference leads to the speech attenuation in the postfilter, which is more crucial for the quality of the enhanced speech. On the other hand, the noise PSD estimation based on the microphone signals in [16,17] essentially estimates the PSD of the noise in the microphone signals, which differs from the PSD of the noise in the beamformer output. In [15], the noise PSD at the beamformer output is estimated from the PSD of the microphone signals, room transfer function, and noise coherence matrix, and a two-step approach to estimate the Wiener postfilter is proposed based on the maximum likelihood approach and the Bayesian refinement. While it provides a novel mathematical framework to estimate the noise PSD and obtain the postfilter, it is assumed that the noise coherence matrix is known in advance. There have been several approaches to apply those filters using dual channel noise PSD estimators without applying beamformers [29,30,31,32]. Among them, Nelke et al. [29] employ a statistical model-based single-channel noise PSD estimator [36] for low-frequency bins and a coherence-based dual channel noise PSD estimator for high-frequency bins, as the coherences are not discriminative for low frequencies. This method can be applied to the estimation of the noise PSD for the postfilter, but the coherence-based dual channel noise PSD estimator in [29] can only estimate the noise PSD in the microphone signals, which will be higher than the noise PSD in the beamformer output.

In this paper, we propose a postfilter for dual channel speech enhancement combining a statistical model-based single-channel noise estimator and a coherence-based dual channel estimator with a SPP. Specifically, we model the coherence-based *a posteriori* SPP, and combine it with the statistical model-based SPP [36]. We then derive the coherence-based dual channel noise PSD estimator considering the speech presence uncertainty and the difference between the noise PSDs in the microphone signals and the beamformer output. Finally, the spectral gain function of the postfilter is computed by utilizing the noise PSD estimate and *a posteriori* SPP based on both the statistical models and the coherences.

## 2. System Overview and Review of SPP-Based Noise Estimation

### 2.1. Problem Formulation and System Overview

Assuming that two microphones capture the desired speech along with the uncorrelated additive noise, the two microphone signals in the STFT domain in a vector form, $Z\left(l,k\right)={\left[Z_{1}\left(l,k\right),Z_{2}\left(l,k\right)\right]}^{T},$ with a time index *l* and a frequency index *k*, can be written as

(1) $$\begin{array}{cc} Z(l,k) & =g\left(l,k\right)S_{1}\left(l,k\right)+V\left(l,k\right)\\ \; & =S(l,k)+V(l,k),\;\;\;\;1\leq l\leq L,1\leq k\leq K \end{array}$$

where $S\left(l,k\right)={\left[S_{1}\left(l,k\right),S_{2}\left(l,k\right)\right]}^{T}$ is the clean speech at the microphones including early reflections, $V\left(l,k\right)={\left[V_{1}\left(l,k\right),V_{2}\left(l,k\right)\right]}^{T}$ is the additive noise at the microphones including late reverberations, and $g\left(l,k\right)={\left[1,g_{2}\left(l,k\right)\right]}^{T}$ is the relative transfer function (RTF) vector. S(l,k) and V(l,k) are assumed to be uncorrelated as in many research works [6,15,19,20,22]. The microphone signals are processed by a filter-and-sum adaptive beamformer $W^{H}\left(l,k\right)=\left[W_{1}^{*}\left(l,k\right),W_{2}^{*}\left(l,k\right)\right]$ to produce the beamformer output $Y\left(l,k\right)=W^{H}\left(l,k\right)Z\left(l,k\right)$. Y(l,k) can be considered to be the sum of the filtered speech X(l,k) and the residual noise N(l,k), which are assumed to be mutually uncorrelated as

(2) $$\begin{array}{cc} Y(l,k) & =W^{H}\left(l,k\right)Z\left(l,k\right)\\ \; & =W^{H}\left(l,k\right)S\left(l,k\right)+W^{H}\left(l,k\right)V\left(l,k\right)\\ \; & ≜X(l,k)+N(l,k). \end{array}$$

**Figure 1:**
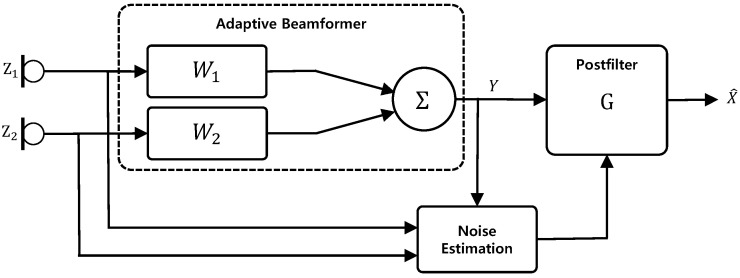
General block diagram of a dual channel speech enhancement system with a beamformer and a postfilter.

The goal of the postfilter is usually to estimate X(l,k) from Y(l,k) with the help of Z(l,k), although X(l,k) may contain speech distortion to an extent. The output of the postfilter is given as

(3) $$\hat{X}\left(l,k\right)=G\left(l,k\right)Y\left(l,k\right)$$

in which G(l,k) is the spectral gain of the postfilter. The block diagram of a dual channel speech enhancement system with an adaptive beamformer and a postfilter is illustrated in Figure 1.

### 2.2. Single-Channel Noise PSD Estimator Based on Speech Presence Probability

In [36], a statistical model-based single-channel noise PSD estimation using a fixed a priori signal-to-noise ratio (SNR) for speech presence is proposed. Under the assumption that the speech and noise STFT coefficients are distributed according to the complex Gaussian distributions with zero means, the likelihood functions for the hypotheses of speech presence $H_{1}$ and speech absence $H_{0}$ are modeled as

(4) $$f\left(Y\left(l,k\right)|H_{0}\right)=\frac{1}{{\hat{\lambda }}_{n_{s}}\left(l,k\right)\pi }exp\left(-\frac{{\left|Y(l,k)\right|}^{2}}{{\hat{\lambda }}_{n_{s}}\left(l,k\right)}\right)$$

(5) $$\begin{array}{cc} f(Y\left(l,k\right)|H_{1})= & \frac{1}{{\hat{\lambda }}_{n_{s}}\left(l,k\right)\left(1+\xi_{H_{1}}\right)\pi }\cdot exp\left(-\frac{{\left|Y(l,k)\right|}^{2}}{{\hat{\lambda }}_{n_{s}}\left(l,k\right)\left(1+\xi_{H_{1}}\right)}\right) \end{array}$$

where $\xi_{H_{1}}$ indicates the fixed a priori SNR, which represents the SNR “if speech were present” [36], and ${\hat{\lambda }}_{n_{s}}\left(l,k\right)$ is the estimate of the noise PSD based on statistical modeling.

According to Bayes’ rule, *a posteriori* SPP $P(H_{1}|Y)$, which is a function of $\left|Y\right|$ and thus denoted as $p_{\left|Y\right|}\left(l,k\right)$ in the next section, is obtained as

(6) $$\begin{array}{c} P\left(H_{1}|\left|Y\right|\right)=p_{\left|Y\right|}\left(l,k\right)={\left\{1+\left(1+\xi_{H_{1}}\right)exp\left(-\frac{{\left|Y\left(l,k\right)\right|}^{2}}{{\hat{\lambda }}_{n_{s}}\left(l-1,k\right)}\frac{\xi_{H_{1}}}{\xi_{H_{1}}+1}\right)\right\}}^{-1} \end{array}$$

where the parameter $\xi_{H_{1}}$ is set to be 15 dB in the experiments, which is obtained by minimizing the total risk of error as in [36], and a priori probability of speech presence $P(H_{1})$ is assumed to be 1/2. To allow the adaptation of the noise PSD estimate when the noise PSD is underestimated, $p_{\left|Y\right|}\left(l,k\right)$ is constrained to be less than 0.99 when smoothed *a posteriori* SPP is higher than 0.99. With *a posteriori* SPP, the noise PSD periodogram in the current frame is estimated as a weighted summation of the noise PSD estimate from the previous frame ${\hat{\lambda }}_{n_{s}}\left(l-1,k\right)$ and the power of the beamformer output signal ${\left|Y(l,k)\right|}^{2}$:

(7) $${\tilde{\lambda }}_{n_{s}}\left(l,k\right)=p_{\left|Y\right|}\left(l,k\right)\cdot {\hat{\lambda }}_{n_{s}}\left(l-1,k\right)+\left(1-p_{\left|Y\right|}\left(l,k\right)\right)\cdot {\left|Y(l,k)\right|}^{2}.$$

Finally, the noise PSD estimate is obtained by recursive smoothing with a smoothing parameter $\alpha_{sm}$ as

(8) $${\hat{\lambda }}_{n_{s}}\left(l,k\right)=\alpha_{sm}{\hat{\lambda }}_{n_{s}}\left(l-1,k\right)+\left(1-\alpha_{sm}\right){\tilde{\lambda }}_{n_{s}}\left(l,k\right).$$

## 3. Postfilter for Dual Channel Speech Enhancement Utilizing Noise Estimation Based on Coherence and Statistical Model

**Figure 2:**
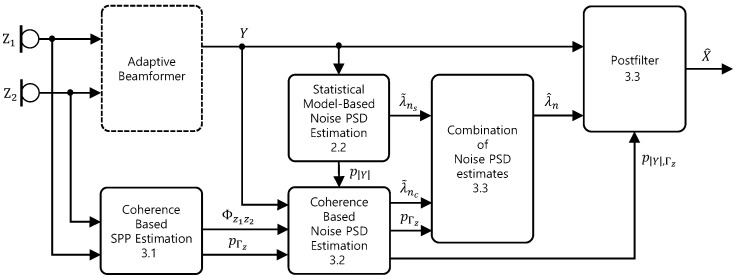
Block diagram of the dual channel speech enhancement system employing the proposed postfilter.

We propose a postfilter for dual channel speech enhancement, combining a statistical model-based single-channel noise estimate and a coherence-based dual channel noise estimate. Assuming that the phase of the coherence between two microphone signals, which is the same as the phase difference between them in the short-time Fourier transform (STFT) domain, is already exploited well by the beamformer, we focus on the magnitude of the coherence as spatial information in the proposed postfilter. Firstly, we model the coherence-based *a posteriori* SPP as a simple function of the magnitude of the coherence, and combine it with the SPP based on the statistical modeling of the beamformer in (6). Then, we derive a dual channel noise PSD estimator for speech presence periods based on coherence, and obtain a noise PSD estimate considering speech presence uncertainty utilizing the *a posteriori* SPP. The final noise PSD estimate is constructed by combining the coherence-based estimate and the statistical model-based single-channel estimate utilizing the coherence-based *a posteriori* SPP. The OM-LSA gain function is used as the postfilter, utilizing the combined noise estimates and the combined *a posteriori* SPP. The block diagram of the proposed method is presented in Figure 2.

### 3.1. Modeling of a Posteriori SPP Based on Coherence

One of the spatial properties that may be used to distinguish signals with different spatial characteristics is the coherence. For two microphone signals $Z_{1}$ and $Z_{2}$, the coherence between them is defined as

(9) $$\Gamma_{z}\left(l,k\right)=\frac{\Phi_{z_{1}z_{2}}\left(l,k\right)}{\sqrt{\Phi_{z_{1}z_{1}}\left(l,k\right)\Phi_{z_{2}z_{2}}\left(l,k\right)}}$$

where $\Phi_{z_{1}z_{2}}$ is the cross PSD of $Z_{1}$ and $Z_{2}$ and $\Phi_{z_{1}z_{1}}$ and $\Phi_{z_{2}z_{2}}$ are the auto PSDs, which can be estimated by temporal smoothing. While the phase of the coherence is related to the inter-channel time difference of arrival for a single directional signal, the magnitude of the coherence is related to how many signals from point sources and image sources accounting for reflections are mixed in the corresponding time–frequency bin. For example, any signals from a point source without reverberation show the magnitude of coherence $\left|\Gamma_{z}\left(l,k\right)\right|$ to be 1. Another useful example is the spherically isotropic or diffuse noise, for which the coherence function can be derived as [38]

(10) $$\Gamma_{z}^{diffuse}\left(l,k\right)=sinc\left(\frac{2\pi kf_{s}d_{mic}}{2Kc}\right)$$

where 2K is the size of the discrete Fourier transform (DFT), $f_{s}$ is the sampling frequency, $d_{mic}$ is the distance between microphones, and *c* is the speed of sound. As the interchannel phase differences, which are the phases of the coherences, are already exploited in the beamformers, we focus on the magnitudes of coherences in the postfilter to utilize complementary information on the spatial characteristics. It is also noted that the directional interferences are taken care of in the adaptive beamformers, and therefore, diffuse noises may be the main obstacles that remain in the beamformer output, which can be effectively discriminated from desired speech using the magnitude of coherence, except low frequencies.

In this paper, it is assumed that the target speaker is located closer to a microphone array than other point sources generating directional interferences. As the distance between a sound source and microphones increases, the magnitude of the coherence decreases due to reverberation. In this regard, the magnitude of the coherence would be high if speech is present in that time–frequency bin, and low when only directional interferences or diffuse noises exist. In this paper, we model the coherence-based *a posteriori* SPP as a simple function of the magnitude of the coherence. As the magnitudes of the coherence in individual time–frequency bins may be vulnerable to the local SNR and reverberation and are not discriminative enough in the low-frequency bins, it is beneficial to aggregate the coherences in all frequency bins to determine frame-wise voice activity and apply separate functions to model *a posteriori* SPP depending on the voice activity. Let $\Gamma_{z}^{\left(f\right)}\left(l\right)=\frac{1}{K}\sum_{k=1}^{K}\left|\Gamma_{z}\left(l,k\right)\right|$ be the frame-wise coherence measure to decide voice activity. The *a posteriori* SPP based on the coherence, $P(H_{1}|\Gamma_{z})$ or $p_{\Gamma_{z}}\left(l,k\right)$, is modeled as

(11) $$\begin{array}{c} P\left(H_{1}|\Gamma_{z}\right)=p_{\Gamma_{z}}\left(l,k\right)=\left\{\begin{array}{cc} \alpha \cdot \left|\Gamma_{z}\left(l,k\right)\right|+\;\alpha_{min} & \;\mathrm{if}\;\Gamma_{z}^{\left(f\right)}\left(l\right)>\eta \\ \beta \cdot \left|\Gamma_{z}\left(l,k\right)\right| & \mathrm{otherwise} \end{array}\right. \end{array}$$

where η is the threshold to apply different functions, and α, $\alpha_{min}$ and β are experimentally determined constants between 0 and 1 with $\alpha +\alpha_{min}\leq 1$. For simplicity, the $p_{\Gamma_{z}}\left(l,k\right)$ is designed as a linear combination of $\left|\Gamma_{z}\left(l,k\right)\right|$ and 1 or 0 for speech presence or absence, respectively. This coherence-based *a posteriori* SPP is used in the coherence-based noise PSD estimation introduced in Section 3.2 and the combination of the statistical model-based and coherence-based noise PSD estimates explained in Section 3.3.

### 3.2. Proposed Dual Channel Noise PSD Estimator Based on Coherence

In order to estimate the noise PSD from dual microphone signals, the noise PSDs for high frequencies were derived as a function of the coherences of the speech and noise and the auto- and cross-PSDs of the microphone signals, while the noise PSD in low frequencies were estimated using an SPP computed from the first microphone signal in [29]. In this paper, we formulate the PSD of the residual noise in the beamformer output N(l,k) as a function of the pseudo-coherences of speech and noise considering the difference in the noise PSD in the microphone signals and the beamformer output and the speech presence uncertainty.

Assuming that the desired speech and the background noise are uncorrelated, the cross PSD of the microphone signals and the PSD of the beamformer output signal can be described as

(12) $$\Phi_{z_{1}z_{2}}\left(l,k\right)=\Phi_{s_{1}s_{2}}\left(l,k\right)+\Phi_{v_{1}v_{2}}\left(l,k\right)$$

(13) $$\Phi_{y}\left(l,k\right)=\Phi_{x}\left(l,k\right)+\Phi_{n}\left(l,k\right)$$

where $\Phi_{s_{1}s_{2}}\left(l,k\right)$ and $\Phi_{v_{1}v_{2}}\left(l,k\right)$ indicate the cross PSD of speech and noise at the first and second microphones, while $\Phi_{x}\left(l,k\right)$ and $\Phi_{n}\left(l,k\right)$ indicate the PSD of speech and noise at the beamformer output, respectively. For speech present regions, we can rewrite the cross PSD of the microphone signals in (12) using the PSDs of the beamformed speech and noise $\Phi_{x}\left(l,k\right)$ and $\Phi_{n}\left(l,k\right)$ in a similar way to [29]

(14) $$\Phi_{z_{1}z_{2}}\left(l,k\right)=\Lambda_{x}\left(l,k\right)\;\Phi_{x}\left(l,k\right)+\Lambda_{n}\left(l,k\right)\;\Phi_{n}\left(l,k\right)$$

where $\Lambda_{x}\left(l,k\right)$ and $\Lambda_{n}\left(l,k\right)$ are the pseudo-coherence of speech and noise considering the effect of beamformers defined as

(15) $$\Lambda_{x}\left(l,k\right)=\frac{\Phi_{s_{1}s_{2}}\left(l,k\right)}{\Phi_{x}\left(l,k\right)}$$

(16) $$\Lambda_{n}\left(l,k\right)=\frac{\Phi_{v_{1}v_{2}}\left(l,k\right)}{\Phi_{n}\left(l,k\right)},$$

which are not the same as the coherence of the speech and noise at the microphones given by

(17) $$\Gamma_{s}\left(l,k\right)=\frac{\Phi_{s_{1}s_{2}}\left(l,k\right)}{\sqrt{\Phi_{s_{1}s_{1}}\left(l,k\right)\Phi_{s_{2}s_{2}}\left(l,k\right)}}$$

(18) $$\Gamma_{v}\left(l,k\right)=\frac{\Phi_{v_{1}v_{2}}\left(l,k\right)}{\sqrt{\Phi_{v_{1}v_{1}}\left(l,k\right)\Phi_{v_{2}v_{2}}\left(l,k\right)}}.$$

Comparing with the original definition of the coherence, we can see that the denominators, the geometric means of the PSDs for speech and noise in two microphone signals in (17) and (18), are replaced by the PSDs of the speech and noise in the beamformer output in (15) and (16).

From Equations (13) and (14), the coherence-based estimate of $\Phi_{n}\left(l,k\right)$ for speech presence, $\Phi_{n|H_{1}}^{coh}\left(l,k\right)$, can be written as a function of $\Phi_{y}\left(l,k\right)$, $\Phi_{z_{1}z_{2}}\left(l,k\right)$, $\Lambda_{x}\left(l,k\right)$ and $\Lambda_{n}\left(l,k\right)$:

(19) $$\Phi_{n|H_{1}}^{coh}\left(l,k\right)=\frac{\Phi_{y|H_{1}}\left(l,k\right)-\frac{\Phi_{z_{1}z_{2}|H_{1}}\left(l,k\right)}{\Lambda_{x}\left(l,k\right)}}{1-\frac{\Lambda_{n}\left(l,k\right)}{\Lambda_{x}\left(l,k\right)}}.$$

As for the speech absent regions, the instantaneous value for the power spectrum of the beamformer output provides the most accurate estimate for the noise PSD in the beamformer output. Therefore, the final dual channel noise PSD based on coherences, ${\tilde{\lambda }}_{n_{c}}\left(l,k\right)$, is given as a linear combination of $\Phi_{n|H_{1}}^{coh}\left(l,k\right)$ and ${\left|Y\left(l,k\right)\right|}^{2}$ in which the weights are determined by the *a posteriori* SPP considering both the beamformed signal and the coherence for microphone signals, $p_{|Y|,\Gamma_{z}}\left(l,k\right)=P\left(H_{1}|\left|Y\right|,\Gamma_{z}\right)$, as follows:

(20) $$\begin{array}{cc} {\tilde{\lambda }}_{n_{c}}\left(l,k\right)= & \;\;p_{|Y|,\Gamma_{z}}\left(l,k\right)\cdot {\hat{\Phi }}_{n|H_{1}}^{coh}\left(l,k\right)+\left(1-p_{|Y|,\Gamma_{z}}\left(l,k\right)\right)\cdot {\left|Y(l,k)\right|}^{2}. \end{array}$$

Assuming that both $\left|Y(l,k)\right|$ and $\Gamma_{z}\left(l,k\right)$ would indicate speech presence if speech is present in that time–frequency bin, $P(H_{1}|\left|Y\right|,\Gamma_{z})$ is represented as the product of the *a posteriori* SPP based on the magnitude spectrum of the beamformed signal $P(H_{1}||Y|)$ in (6), and the *a posteriori* SPP based on the coherence $P(H_{1}|\Gamma_{z})$ in (11), i.e.,

(21) $$p_{|Y|,\Gamma_{z}}\left(l,k\right)=p_{\left|Y\right|}\left(l,k\right)\cdot p_{\Gamma_{z}}\left(l,k\right)$$

To evaluate (19), the pseudo-coherences of the speech and noise $\Lambda_{x}\left(l,k\right)$ and $\Lambda_{n}\left(l,k\right)$ need to be estimated in addition to $\Phi_{y|H_{1}}\left(l,k\right)$ and $\Phi_{z_{1}z_{2}|H_{1}}\left(l,k\right)$, which can be obtained by the temporal smoothing of ${\left|Y\left(l,k\right)\right|}^{2}$ and $Z_{1}\left(l,k\right)Z_{2}^{*}\left(l,k\right)$ for speech presence periods. The pseudo-coherences can be estimated in a similar way to the coherence estimation in [29] using the SPP in (21). We omit the frame and frequency indices for brevity. The estimate for noise pseudo-coherence, ${\hat{\Lambda }}_{n}$, is updated with a smoothing parameter $\alpha_{\Lambda }$ during noise-only periods determined by the *a posteriori* SPP $p_{|Y|,\Gamma_{z}}$ as

(22) $${\hat{\Lambda }}_{n}=\alpha_{\Lambda }\cdot {\hat{\Lambda }}_{n,last}+\left(1-\alpha_{\Lambda }\right)\;{\hat{\Lambda }}_{Y|H_{0}},\;\;\mathrm{if}\;p_{|Y|,\Gamma_{z}}<p_{th_{1}}$$

where $p_{th_{1}}$ is a threshold to update ${\hat{\Lambda }}_{n}$, ${\hat{\Lambda }}_{n,last}$ denotes the estimate of noise pseudo-coherence in the frame it was updated lastly, and ${\hat{\Lambda }}_{Y|H_{0}}$ denotes the estimate for the pseudo-coherence of the noisy signal for speech absent regions considering the effect of beamformers defined as

(23) $${\hat{\Lambda }}_{Y|H_{0}}=\frac{{\hat{\Phi }}_{z_{1}z_{2}|H_{0}}}{{\hat{\Phi }}_{y|H_{0}}},$$

in which $\Phi_{y|H_{0}}\left(l,k\right)$ and $\Phi_{z_{1}z_{2}|H_{0}}\left(l,k\right)$ are estimated by the temporal smoothing of ${\left|Y\right|}^{2}$ and $Z_{1}Z_{2}^{*}$ during noise-only periods in a similar way to (22).

The estimation of $\Lambda_{x}$ is not as straightforward as that for $\Lambda_{n}$ because the background noises reside also in the speech-active regions. The pseudo-coherence of the noisy signal for speech present regions, $\Lambda_{Y|H_{1}}$, can be expressed as

(24) $$\begin{array}{cc} \Lambda_{Y|H_{1}} & =\frac{\Phi_{z_{1}z_{2}|H_{1}}}{\Phi_{y|H_{1}}}\\ \; & =\frac{\Phi_{s_{1}s_{2}}+\Phi_{v_{1}v_{2}}}{\Phi_{x}+\Phi_{n}}\\ \; & =\frac{\Phi_{s_{1}s_{2}}}{\Phi_{x}}\;\left(\frac{\Phi_{x}}{\Phi_{x}+\Phi_{n}}\right)+\frac{\Phi_{v_{1}v_{2}}}{\Phi_{n}}\;\left(\frac{\Phi_{n}}{\Phi_{x}+\Phi_{n}}\right)\\ \; & =\Lambda_{x}\;\left(\frac{\gamma }{1+\gamma }\right)+\Lambda_{n}\;\frac{1}{1+\gamma } \end{array}$$

where $\gamma =\frac{\Phi_{x}}{\Phi_{n}}$ is the SNR at the beamformer output, which is estimated using the statistical model-based noise PSD estimate in (8) as

(25) $$\hat{\gamma }=\frac{{\hat{\Phi }}_{y|H_{1}}}{{\hat{\lambda }}_{n_{s}}}-1$$

in which ${\hat{\Phi }}_{y|H_{1}}$ is obtained by the temporal smoothing of beamformer outputs in speech-active periods, i.e., the time–frequency bins with $p_{|Y|,\Gamma_{z}}>p_{th_{2}}$. The left-hand side of (24), $\Lambda_{Y|H_{1}}$, can also be estimated as $\frac{{\hat{\Phi }}_{z_{1}z_{2}|H_{1}}}{{\hat{\Phi }}_{y|H_{1}}}$, in which ${\hat{\Phi }}_{z_{1}z_{2}|H_{1}}$ is obtained in a similar way to ${\hat{\Phi }}_{y|H_{1}}$. Then, the speech pseudo-coherence $\Lambda_{x}\left(l,k\right)$ can be estimated according to (24) using ${\hat{\Lambda }}_{Y|H_{1}}$, $\hat{\gamma }$ in (25), and ${\hat{\Lambda }}_{n}$ in (22) with additional temporal smoothing in the speech presence periods as

(26) $$\begin{array}{cc} {\hat{\Lambda }}_{x}= & \alpha_{\Lambda }\cdot {\hat{\Lambda }}_{x,last}+\left(1-\alpha_{\Lambda }\right)\left[{\hat{\Lambda }}_{Y|H_{1}}\frac{\hat{\gamma }+1}{\hat{\gamma }}-{\hat{\Lambda }}_{n}\frac{1}{\hat{\gamma }}\right],\;\;\mathrm{if}\;p_{|Y|,\Gamma_{z}}>p_{th_{2}}. \end{array}$$

### 3.3. Combining Noise PSD Estimates and Gain Calculation

**Figure 3:**
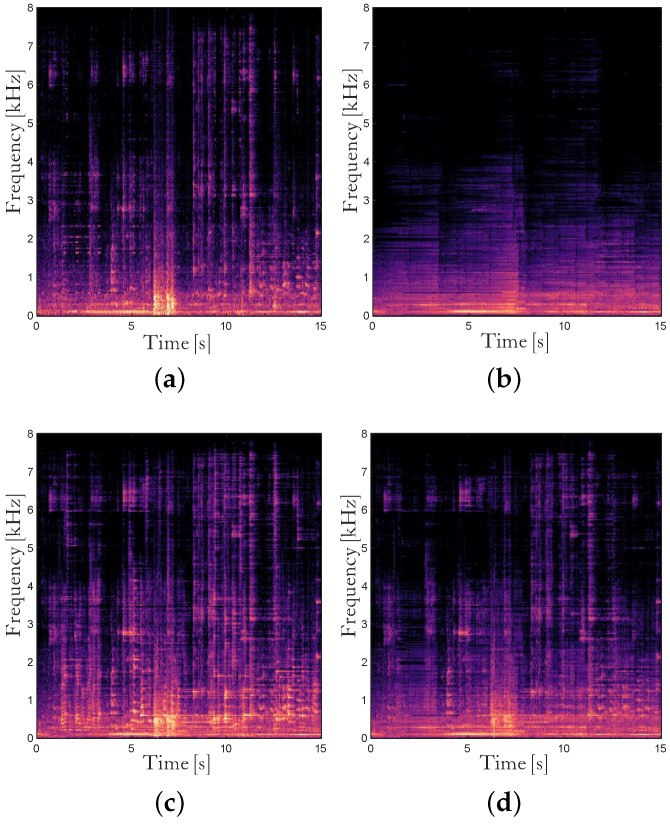
Noise power spectrum and the estimates of it before temporal smoothing for Cafeteria noise at 5 dB SNR in the smaller simulated room. (**a**) True noise power spectrum at the beamformer output, (**b**) the single-channel SPP-based estimate in (7), (**c**) the coherence-based estimate in (20), and (**d**) the combined estimate in (27).

The proposed dual channel noise PSD estimator based on coherence in (20) shows different characteristics from the single-channel SPP-based noise PSD estimator in (7). Figure 3 shows one example of the noise power spectrum in the beamformer output and the estimates of it for Cafeteria noise at 5 dB SNR. The single-channel SPP-based estimate ${\tilde{\lambda }}_{n_{s}}$ in Figure 3b seems to be stable and reliable, while it cannot track abrupt changes in the noise power spectrum. In contrast, the dual channel coherence-based estimate ${\tilde{\lambda }}_{n_{c}}$ in Figure 3c could deal with rapidly changing noises, but occasionally, a certain portion of the speech power spectrum is included in the noise power spectrum estimate. The speech leakage in the noise power spectrum estimate leads to speech distortion when applying the postfilter, and thus is much more critical for the quality of the enhanced speech than the underestimation of the noise statistics. Therefore, we combine two noise power spectrum estimates ${\tilde{\lambda }}_{n_{s}}$ and ${\tilde{\lambda }}_{n_{c}}$ as

(27) $${\tilde{\lambda }}_{n}\left(l,k\right)=p_{\Gamma_{z}}\left(l,k\right)\cdot {\tilde{\lambda }}_{n_{s}}\left(l,k\right)+\left(1-p_{\Gamma_{z}}\left(l,k\right)\right)\cdot {\tilde{\lambda }}_{n_{c}}\left(l,k\right),$$

so that it follows ${\tilde{\lambda }}_{n_{s}}$ in the presence of speech signal and becomes ${\tilde{\lambda }}_{n_{c}}$ when it is certain that speech is absent. It is noted that we use $p_{\Gamma_{z}}$ instead of $p_{|Y|,\Gamma_{z}}$ as the weight to react faster to the speech onsets. ${\tilde{\lambda }}_{n}$ is shown in Figure 3d, which looks similar to the true noise power spectrum at the beamformer output ${\left|N\left(l,k\right)\right|}^{2}$ in Figure 3a compared with ${\tilde{\lambda }}_{n_{s}}$ and ${\tilde{\lambda }}_{n_{c}}$.

The final estimate of the noise PSD ${\hat{\lambda }}_{n}$ is obtained by the temporal smoothing of ${\tilde{\lambda }}_{n}$ with a smoothing parameter $\alpha_{n}$ as

(28) $${\hat{\lambda }}_{n}\left(l,k\right)=\alpha_{n}{\hat{\lambda }}_{n}\left(l-1,k\right)+\left(1-\alpha_{n}\right){\tilde{\lambda }}_{n}\left(l,k\right).$$

Using the noise PSD estimate ${\hat{\lambda }}_{n}$ in (28) and the *a posteriori* SPP $p_{|Y|,\Gamma_{z}}$ in (21), the gain function of the postfilter *G* can be computed as the OM-LSA speech estimator [39]

(29) $$G\left(l,k\right)={\left\{\max(\tilde{G}\left(l,k\right),G_{min})\right\}}^{p_{|Y|,\Gamma_{z}}\left(l,k\right)}\cdot G_{min}^{1-p_{|Y|,\Gamma_{z}}\left(l,k\right)}$$

where $G_{min}$ indicates a minimum value for the gain in speech absent periods and $\tilde{G}\left(l,k\right)$ is the spectral gain function of the minimum mean-square error short-time log spectral amplitude (MMSE-LSA) estimator given by [40]

(30) $$\tilde{G}\left(l,k\right)=\frac{\xi (l,k)}{1+\xi (l,k)}\exp\left[\frac{1}{2}\int_{v(l,k)}^{\infty }\frac{e^{-t}}{t}dt\right]$$

where v(l,k)≜γ(l,k)ξ(l,k), $\xi \left(l,k\right)≜E{\left|X(l,k)\right|}^{2}/{\hat{\lambda }}_{n}\left(l,k\right)$ indicates the a priori SNR at the beamformer output estimated using a decision-directed (DD) approach [5], and $\gamma \left(l,k\right)≜{\left|Y(l,k)\right|}^{2}/{\hat{\lambda }}_{n}\left(l,k\right)$ is the *a posteriori* SNR.

## 4. Experimental and Results

### 4.1. Experimental Configurations

To demonstrate the performance of the proposed coherence and statistical model-based dual channel noise PSD estimator and the postfilter, we simulated two rooms of dimensions 6.7 m × 6.1 m × 2.9 m and 9 m × 7.5 m × 3.5 m using the image method [41,42]. The reverberation times were ${\mathrm{RT}}_{60}=300$ ms and ${\mathrm{RT}}_{60}=500$ ms, respectively. The microphones were located at (3 m, 3 m, 1.5 m) and (3.14 m, 3 m, 1.5 m) for both of the rooms, which corresponded to the form factor of the modern smartphones in the landscape orientation. We assumed the “hand-held handsfree” scenario [43] in which the desired speaker was located at the broadside of the microphone array, 0.4 m away from the center of the microphones.

In addition, we also utilized a real-recorded room impulse response (RIR) from the multi-channel impulse response database (MIRD) [44] with the room dimensions of 6 m × 6 m × 2.4 m and the ${\mathrm{RT}}_{60}$ of 360 ms. The desired speaker was assumed to be located 1 m away from the center of two microphones at the broadside direction. The 1 m distance was not a typical one for the “hand-held handsfree” use cases and was not favorable to the proposed method utilizing the coherence information, but we could not find a more suitable real-recorded RIR database. The distance between the two microphones we utilized was 14 cm as in the simulated RIR cases, in accordance with the size of the recent smartphones.

Twelve utterances from the TIMIT database [45] were used as desired speech signals, and the Cafeteria, Crossroad, Kindergarten 1, Pub, Train Station, Callcenter, and Mensa noises from ES 202 396-1 [46] were used to generate diffuse noises using the arbitrary noise field generator [47]. The SNRs for diffuse noises were −5, 0, 5, 10, and 15 dB. The signals were sampled at 16 kHz, and 512-point STFT was applied to the 32 ms of windowed signal with 20 ms frame shift, in which the Tukey window with the cosine fraction of 75% was adopted.

We compared the performance of the proposed postfilter to those of the postfilter using the TBRR-based multichannel noise PSD estimator [21], which is denoted as *TBRR*, and the one adopting the SPP-based single-channel noise estimator introduced in Section 2.2 [36], which is denoted as *Single-SPP*. Although there have been several recent research studies on better spatial filtering [9,10], not much effort has been devoted to improve the postfilters for the spatial filtering recently, except for the deep learning-based approaches. Deep learning-based postfilters using single-channel [24,25] or multichannel information [23,27,28] have been proposed, but these approaches often require high computational complexity and large training datasets.

The general transfer function-generalized sidelobe canceler (TF-GSC) [21] was used as the adaptive beamformer with the same parameter values as in [21]. The OM-LSA speech estimator in (29) and (30) was employed as a postfilter for all three methods, in which different SPP and noise PSD estimators were adopted. The parameter values for the proposed method used for the experiments are summarized in Table 1. The smoothing parameters to compute $\Phi_{y|H_{0}}$ and $\Phi_{z_{1}z_{2}|H_{0}}$ in (23) were 0.4, while those to obtain $\Phi_{y|H_{1}}$ and $\Phi_{z_{1}z_{2}|H_{1}}$ that were used to compute ${\hat{\Lambda }}_{Y|H_{1}}$ and $\hat{\gamma }$ were 0.7. The parameters for the compared methods were selected to maximize the average PESQ score for the enhanced speech, which were the same as the values in the original papers, except $\alpha_{\lambda }$ was 0.8 instead of 0.85 in [21].

As the performance measure for the noise PSD estimation accuracy, we used the segmental logarithmic error (LogErr) defined by

(31) $$\mathrm{LogErr}=\frac{1}{KL}\sum_{k=1}^{K}\sum_{l=1}^{L}\left|10\;log_{10}\left[\frac{\lambda_{n}\left(l,k\right)}{{\hat{\lambda }}_{n}\left(l,k\right)}\right]\right|$$

where $\lambda_{n}\left(l,k\right)$ indicates the true noise PSD at the beamformer output obtained by processing the noise with the TF-GSC computed for the noisy input and applying temporal smoothing in (28). The logarithmic error can be represented as a summation of the overestimation error ${\mathrm{LogErr}}_{\mathrm{ov}}$ and the underestimation error ${\mathrm{LogErr}}_{\mathrm{un}}$ of the noise PSD, which are defined as [36]

(32) $${\mathrm{LogErr}}_{\mathrm{ov}}=\frac{1}{KL}\sum_{k=1}^{K}\sum_{l=1}^{L}\left|\min\left(0,10\;log_{10}\left[\frac{\lambda_{n}\left(l,k\right)}{{\hat{\lambda }}_{n}\left(l,k\right)}\right]\right)\right|$$

(33) $${\mathrm{LogErr}}_{\mathrm{un}}=\frac{1}{KL}\sum_{k=1}^{K}\sum_{l=1}^{L}\max\left(0,10\;log_{10}\left[\frac{\lambda_{n}\left(l,k\right)}{{\hat{\lambda }}_{n}\left(l,k\right)}\right]\right).$$

The ${\mathrm{LogErr}}_{\mathrm{ov}}$ may indicate the degree of speech attenuation caused by the postfilter, while ${\mathrm{LogErr}}_{\mathrm{un}}$ would be related to the amount of residual noises. As for the speech enhancement performance, the ITU-T Recommendation P.862.2 wideband perceptual evaluation of speech quality (PESQ) [48] scores and the segmental SNR (SSNR) improvement were evaluated. The SSNR is defined as

(34) $$\mathrm{SSNR}=\frac{10}{\left|L\right|}\sum_{l\in L}\log_{10}\frac{\sum_{n=1}^{N}s^{2}\left(lN+n\right)}{\sum_{n=1}^{N}{\left(\hat{s}\left(lN+n\right)-s\left(lN+n\right)\right)}^{2}}$$

where N=160, L is the set of speech active segments and s(l), and $\hat{s}\left(l\right)$ indicate the clean speech signal and the estimate of it in the time domain, respectively.

As the proposed method relies on the coherence information and the coherences for the directional interferences would be higher than those for the diffuse noises, the performance for the proposed method may deteriorate in the presence of directional interference. To demonstrate that the proposed system does not show inferior performance to the previous approaches in the presence of both diffuse noise and directional interference, we conducted an additional experiment on the smaller simulated room. Directional interference was constructed by the image method [41,42] using three utterances from the Wall Street Journal (WSJ0) dataset [49] in which the noise source was located at $-30^{\circ }$ from the broadside direction, 0.8 m away from the center of the microphones. The signal-to-interference ratios (SIRs) for the directional interferences were 0, 5, and 10 dB.

### 4.2. Experimental Results

**Figure 4:**
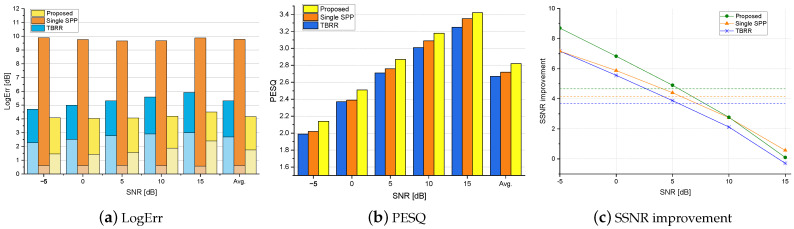
The logarithmic errors, PESQ scores, and SSNR improvements for the proposed and competing postfilters averaged over all types of diffuse noise in various SNRs for the smaller simulated room with the ${\mathrm{RT}}_{60}$ of 300 ms. The lower and upper bars in the LogErr plot represent the overestimation and underestimation errors, respectively. The average scores are shown as the rightmost bars (**a**,**b**) or dashed lines (**c**).

**Figure 5:**
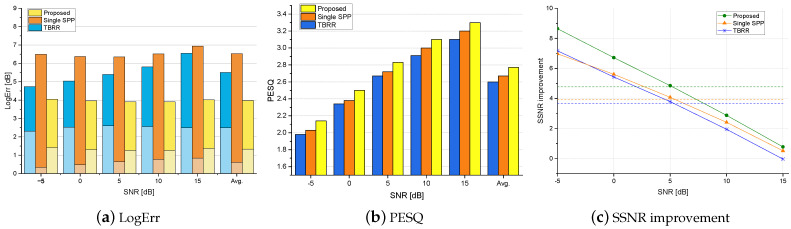
The logarithmic errors, PESQ scores, and SSNR improvements for the proposed and competing postfilters averaged over all types of diffuse noise in various SNRs for the larger simulated room with the ${\mathrm{RT}}_{60}$ of 500 ms. The lower and upper bars in the LogErr plot represent the overestimation and underestimation errors, respectively. The average scores are shown as the rightmost bars (**a**,**b**) or dashed lines (**c**).

**Figure 6:**
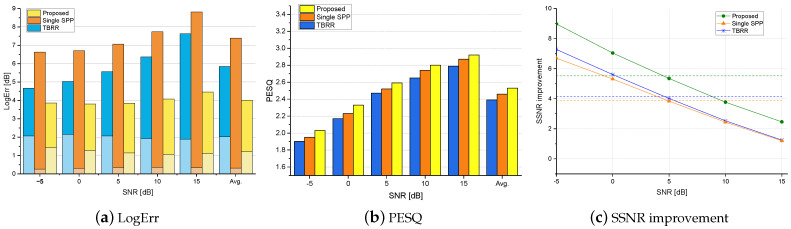
The logarithmic errors, PESQ scores, and SSNR improvements for the proposed and competing postfilters averaged over all types of diffuse noise in various SNRs for the real-recorded RIR database with the ${\mathrm{RT}}_{60}$ of 360 ms. The lower and upper bars in the LogErr plot represent the overestimation and underestimation errors, respectively. The average scores are shown as the rightmost bars (**a**,**b**) or dashed lines (**c**).

**Figure 7:**
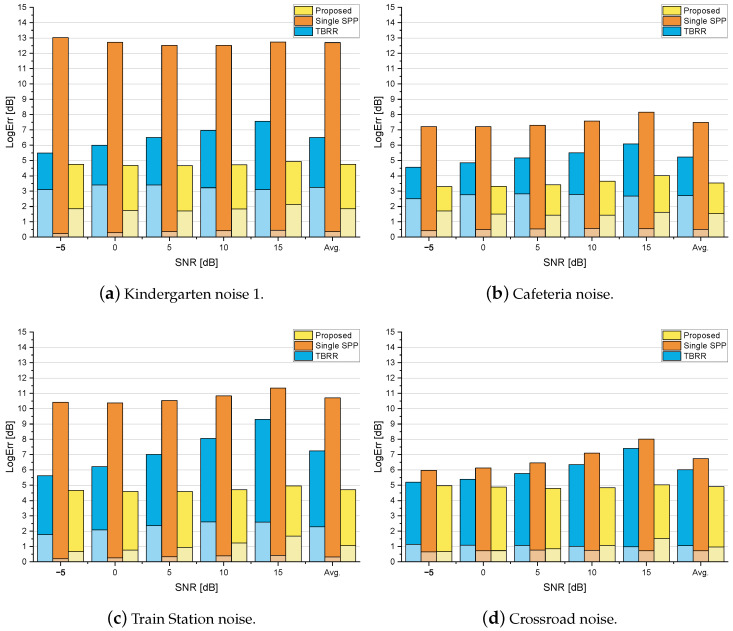
The logarithmic errors of the proposed and competing noise PSD estimators for four types of diffuse noises averaged over three room conditions depending on the input SNR. The lower and upper bars indicate the overestimation and underestimation errors, respectively.

Figure 4, Figure 5 and Figure 6 show the logarithmic errors, PESQ scores, and SSNR improvements for the proposed and compared methods averaged over seven types of diffuse noise for various SNRs. Figure 4 and Figure 5 are for the simulated rooms with the ${\mathrm{RT}}_{60}$ of 300 ms and 500 ms, respectively, and the distance to the desired speaker of 40 cm. Figure 6 is for the real-recorded RIR with the ${\mathrm{RT}}_{60}$ of 360 ms and the distance to the desired speaker of 1 m. The performance results averaged over all SNR conditions are shown as the rightmost bar graphs or dashed lines. As for the noise PSD estimation accuracy in terms of LogErr, the proposed method exhibited the lowest LogErr for all cases. The *TBRR* showed lower LogErr than the *Single-SPP*, but the ${\mathrm{LogErr}}_{\mathrm{ov}}$ for the *TBRR* was higher. Compared with the *Single-SPP* [36], the noise underestimation errors for the proposed method were reduced, while the noise overestimation errors were slightly increased. It implies that the proposed coherence-based noise PSD estimator in (20) could track abrupt change in the noise power spectrum as illustrated in Figure 3c, which resulted in the final noise PSD estimate in (28) close to the true PSD. On the other hand, it can be found that the noise overestimation of the *TBRR* [21] was higher than that of the proposed method, which would be more crucial to the perceptual quality of enhanced speech. Figure 7 shows the logarithmic errors for two highly nonstationary noises, the Kindergarten noise 1 and the Cafeteria noise, and two more stationary noises, the Train Station noise and Crossroad noise, for each SNR averaged over three room conditions. The proposed method marked the lowest LogErr for all noise types and SNRs. The *TBRR* [21] tended to overestimate the noise PSD more in the presence of highly nonstationary noises, although it was originally proposed to tackle relatively nonstationary diffuse noises. The TBRR in the speech active region was occasionally underestimated in the presence of highly nonstationary noises since the transient power in the noise reference of the TF-GSC became high, leading to a low *a posteriori* SPP and overestimation of the noise PSD. The ${\mathrm{LogErr}}_{\mathrm{un}}$ of the *Single-SPP* increased as the noise became more nonstationary, i.e., from the Crossroad noise in Figure 7d through the Cafeteria noise in Figure 7b to the Kindergarten 1 noise in Figure 7a, as the single-channel noise estimation would regard highly nonstationary noise as speech. It is noted that the Train Station noise in Figure 7c is relatively stationary on average but includes occasional nonstationary events, and thus it did not show a clear tendency among the other three noises. The ${\mathrm{LogErr}}_{\mathrm{ov}}$ for the proposed method also increased for more nonstationary noises, maybe because the local SNRs in specific time–frequency bins could be very low in highly nonstationary noises for the same input SNR, which led to low *a posteriori* SPP and noise PSD overestimation.

**Figure 8:**
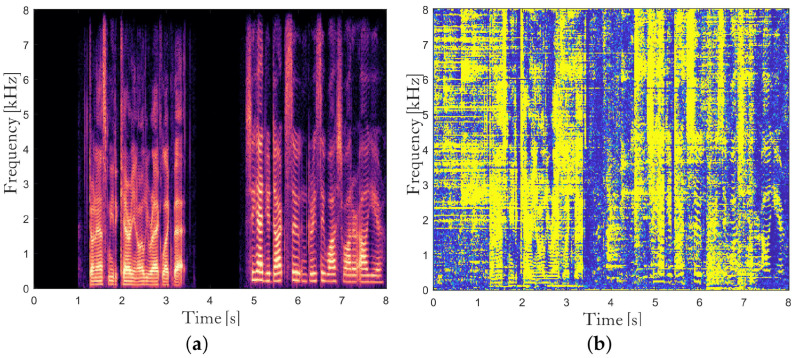

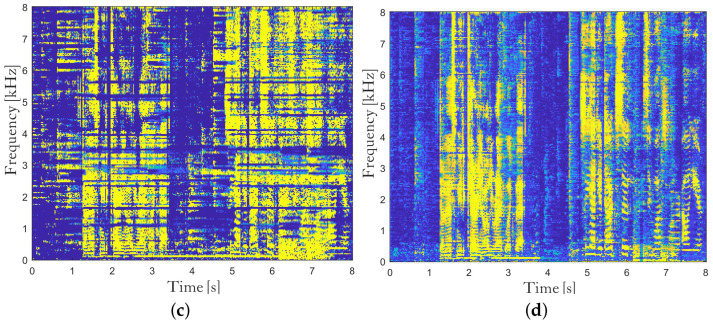
The spectrogram of the desired clean speech (**a**) and the *a posteriori* SPPs estimated by the *Single-SPP* [36] (**b**), the *TBRR* [21] (**c**), and the proposed method (**d**) for two utterances in the Cafeteria noise at 5 dB SNR in the smaller simulated room with the ${\mathrm{RT}}_{60}$ of 300 ms.

Figure 8 presents the spectrogram of the desired clean speech and the *a posteriori* SPPs estimated by the proposed and competing methods for the concatenation of two utterances in the Cafeteria noise at 5 dB SNR for the smaller simulated room. The *Single-SPP* [36] shows overestimation of the *a posteriori* SPP in many TF bins in Figure 8b, as it cannot easily discriminate the noise onset from the speech onset. The noise onset causes overestimation of the SPP in the speech absent region, which leads to the underestimation of the noise PSD, and the underestimated noise PSD in turn results in the overestimation of the SPP for the upcoming frames. The *TBRR* [21] makes blue horizontal lines in Figure 8c, in which the inaccurate estimation of the acoustic transfer function in the TF-GSC brings about the leakage of the speech to the noise references, which lowers the TBRR together with the transient noises and then results in low *a posteriori* SPP. In contrast, both the coherence-based and statistical model-based noise PSD estimators in the proposed method are not tightly coupled with the performance of the beamformer, and thus occasional failure of the TF-GSC does not affect the postfilter critically. We can also see that the proposed method shows better speech onset detection and a clearer harmonic structure.

The speech enhancement performances for the proposed and compared postfilters in the presence of diffuse noises are shown in the PESQ scores and the SSNR improvements in Figure 4, Figure 5 and Figure 6. Since the ${\mathrm{LogErr}}_{\mathrm{ov}}$ is lower for the *Single-SPP* compared with that for the *TBRR* although the LogErr for the *TBRR* is lower, the average PESQ scores are higher for the *Single-SPP* than those for the *TBRR*. The proposed postfilter with new noise PSD and *a posteriori* SPP estimators result in the highest PESQ scores for all SNRs and all room conditions. The PESQ score for the proposed method averaged over all room and noise conditions is 2.71, 0.09 higher than that for the second best one, *Single-SPP*, with the *p*-value of 0.014. As the SNR increases, the PESQ score improvement of the proposed method over the *Single-SPP* decreases, whereas the difference between the PESQ scores for the *Single-SPP* and the *TBRR* increases. It may be because the *Single-SPP* tends to underestimate the noise PSD, resulting in less speech attenuation and more residual noise, which is less crucial in the high SNR environments where the noise power is low from the start. In terms of the SSNR improvement, the proposed postfilter demonstrates the best performance for all SNRs and all room conditions except the 15 dB SNR in the smaller simulated room. As the power of the noise in comparison with the speech power is small for the 15 dB SNR, the power of the residual noise is small with a similar ${\mathrm{LogErr}}_{\mathrm{un}}$, and thus the SSNR improvement could become high for the *Single-SPP* with low ${\mathrm{LogErr}}_{\mathrm{ov}}$ and high ${\mathrm{LogErr}}_{\mathrm{un}}$ as in the PESQ scores. In the same regard, the SSNR improvement for the *Single-SPP* subtracted by that for the *TBRR* increases with the SNR. Although the PESQ scores for the *Single-SPP* are higher than those for the *TBRR* in all conditions, the SSNR improvements for these approaches are comparable, possibly because the speech attenuation caused by the noise PSD overestimation is more critical for the PESQ scores compared with the SSNR improvement. The SSNR improvements for the *TBRR* are higher than those for the *Single-SPP* under the conditions where the ${\mathrm{LogErr}}_{\mathrm{ov}}$ for the *TBRR* are not high.

**Figure 9:**
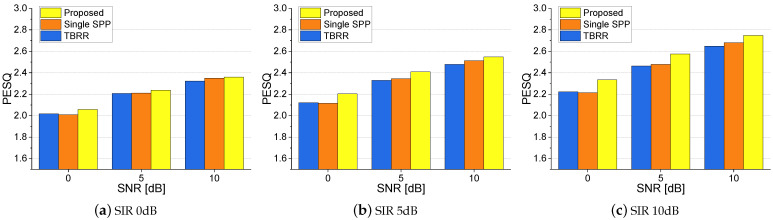
Average PESQ scores for the proposed and competing noise PSD estimators with both the diffuse noises and directional interferences in various SNRs and SIRs in the smaller simulated room.

As the *a posteriori* SPP based on the coherence in (11) and the coherence-based noise PSD estimator in (20) relies on the difference of the coherences for the desired speech and noise, the performance of the proposed method in the presence of directional interference, which would have a higher magnitude coherence, than the diffuse noises may be questionable. To ensure that the performance of the speech enhancement does not degrade by the adoption of the proposed coherence-based SPP and noise PSD estimators in the presence of directional interferences, we conducted another set of experiments with both diffuse noises and directional interferences in various SNRs and SIRs in the smaller simulated room with the ${\mathrm{RT}}_{60}$ of 300 ms. Figure 9 presents the average PESQ scores for the proposed method and competing noise PSD estimators with various SNRs and SIRs. Although the performance improvement over other approaches is reduced to 0.06 on average, the *p*-value over the *Single-SPP* is 0.000055. We can verify that the proposed method performs slightly better than other approaches even when directional interferences disrupt the coherence-based estimators.

## 5. Conclusions

In this work, we have proposed a postfilter for dual channel speech enhancement utilizing *a posteriori* SPP and noise PSD estimators based on both coherence and statistical models. We have modeled the *a posteriori* SPP as a function of the magnitude of the coherence between dual microphone signals and integrated it with the statistical model-based SPP, which is then utilized for the noise PSD estimation and the OM-LSA gain function. The coherence-based noise PSD estimator is derived considering the difference between the noises in the microphone signals and the beamformer output and the speech presence uncertainty explicitly, and is combined with the SPP-based single-channel noise PSD estimator using the proposed coherence-based SPP. Experimental results show that the proposed method leads to more accurate estimation of the noise PSD and better speech enhancement in terms of the logarithmic error, PESQ scores, and SSNR improvement under the presence of various types of diffuse noise in three room conditions for the “hand-held handsfree” scenario. It is also demonstrated that the proposed method slightly outperforms competing methods in the presence of both diffuse noises and directional interferences even though the proposed approach utilizes the coherence information.

## Figures and Tables

**Table 1:** Parameters for the proposed statistical model and coherence-based postfilter.

$\alpha_{\mathrm{sm}}$	α	$\alpha_{\min}$	β	η	$\alpha_{\Lambda }$	$\alpha_{n}$	$p_{{\mathrm{th}}_{1}}$	$p_{{\mathrm{th}}_{2}}$	$G_{\min}$
0.8	0.75	0.2	0.5	0.377	0.95	0.8	0.1	0.6	−9 dB

## Data Availability

Data are contained within the article.
